# Supplementary material for: Extended supercooled storage of red blood cells
Source: Commun Biol. 2024 Jun 24;7:765. doi: 10.1038/s42003-024-06463-4 (PMC11196592; doi:10.1038/s42003-024-06463-4)
Supplement: Supplementary file 2 — Supplementary Information [file 42003_2024_6463_MOESM2_ESM.pdf]

Supplementary Information

Extended supercooled storage of red blood cells

Ziya Isiksacan[1,2], Nishaka William[3], Rahime Senturk[1,2,4], Luke Boudreau[1,2], Celine Wooning[1,2,5], Emily Castellanos[1,2,6], Salih Isiksacan,[1,2,7] Martin L. Yarmush[1,2,8], Jason P. Acker[3,9]*, and O. Berk Usta[1,2]*

[1]Center for Engineering in Medicine and Surgery, Massachusetts General Hospital, Harvard Medical School, Boston, MA, United States [2]Shriners Children's, Boston, MA, United States [3]Department of Laboratory Medicine and Pathology, University of Alberta, Edmonton, AB, Canada [4]Department of Chemical Engineering, Eindhoven University of Technology, Eindhoven, Netherlands [5]Department of Human Biology, Scripps College, Claremont, CA, United States [6]Department of Psychology, Amherst College, Amherst, MA, United States [7]Department of Electrical-Electronics Engineering, Bilkent University, Ankara, Turkey [8]Department of Biomedical Engineering, Rutgers University, Piscataway, NJ, United States [9]Innovation and Portfolio Management, Canadian Blood Services, Edmonton, AB, Canada

* Corresponding authors: Jason P. Acker (jacker@ualberta.ca) and O. Berk Usta (ousta@mgh.harvard.edu)

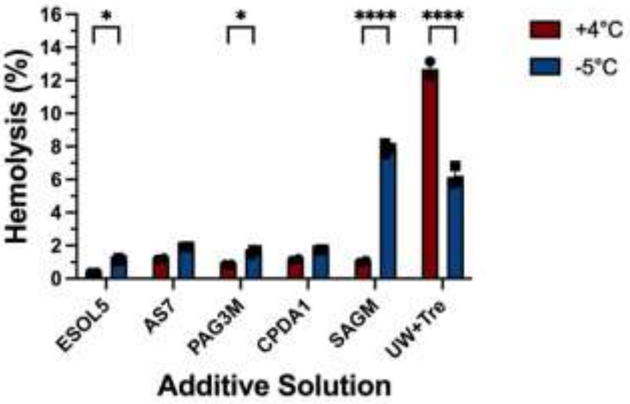

Supplementary Fig. 1 Initial screening of 3 next-generation, 2 standard additive solutions, and 5% trehalose-supplemented UW (UW+Tre) for hemolysis at hypothermic (+4 °C) and supercooled (-5 °C) conditions. The samples were stored for 4 weeks. Samples stored in E-Sol 5, AS-7, PAG3M, and CPDA-1 experienced similar hemolysis levels between 1-2% at -5 °C. SAGM and UW+Tre caused more than 5% hemolysis at -5 °C. All solutions resulted in similar and close to 1% hemolysis at +4 °C, except UW+Tre with 12% hemolysis. Data represent mean ± standard deviation from one biological replicate (N=1) and two technical replicates (n=2) for +4 °C and three technical replicates (n=3) for -5 °C. A two-way analysis of variance (ANOVA) followed by Sidak's *post hoc* test was performed to evaluate significant differences between conditions: *p < 0.05; **p < 0.01; ***p < 0.001; ****p < 0.0001. For clarity of the plots, comparisons were made for each solution across the two temperatures.

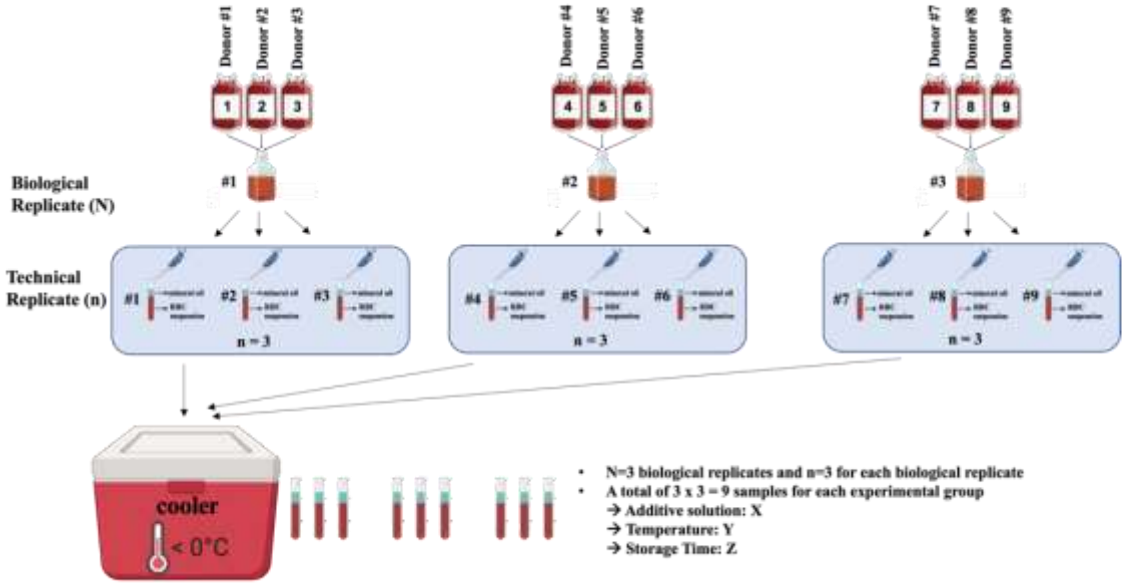

Supplementary Fig. 2 The study design for the experiments. Experiments were conducted from 3 biological replicates (N=3) and three technical replicates for each biological replicate. Each biological replicate was from a pool of 3 donor samples.

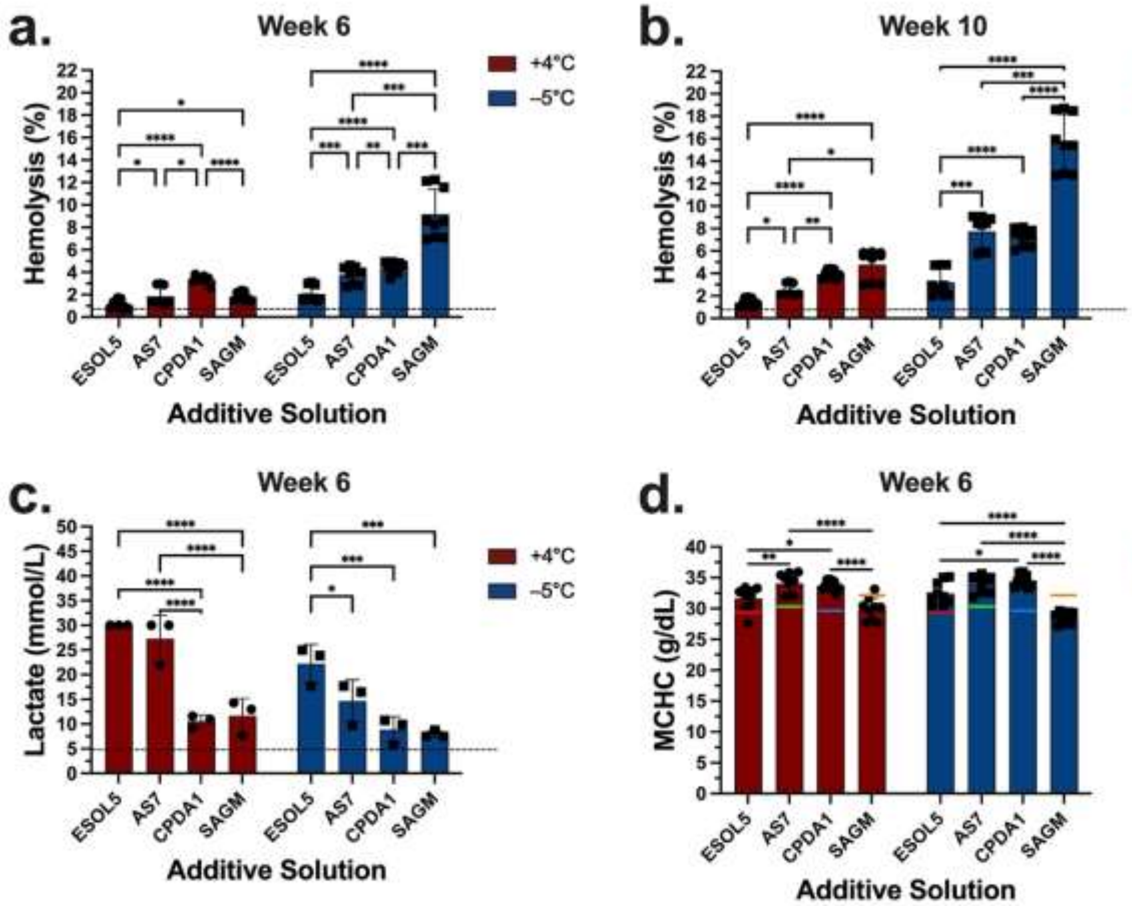

Supplementary Fig. 3 Comparison of E-Sol 5, AS-7, CPDA-1, and SAGM for hemolysis, lactate, and MCHC at +4 °C and -5 °C after 6 and 10 weeks of storage. **a.** The samples were stored for 6 weeks. **b.** The samples were stored for 10 weeks. **c.** Lactate levels were higher for E-Sol 5 and AS-7 at both temperatures. **d.** The samples stored in SAGM showed an increased MCHC at both temperatures. Colored lines represent the initial levels. Dashed lines represent the mean day-1 levels following 3x washing, overnight storage at +4 °C, and final 1x washing in respective solutions. Data represent mean ± standard deviation from 3 biological replicates (N=3) and three technical replicates (n=3, n=1-3 for lactate) for each biological replicate. Each biological replicate was from a pool of 3 donor samples. A two-way analysis of variance (ANOVA) followed by Tukey's *post hoc* test was performed to evaluate significant differences between conditions: *p < 0.05; **p < 0.01; ***p < 0.001; ****p < 0.0001. Comparisons were shown across the solutions at each temperature. MCHC: mean cell hemoglobin concentration.

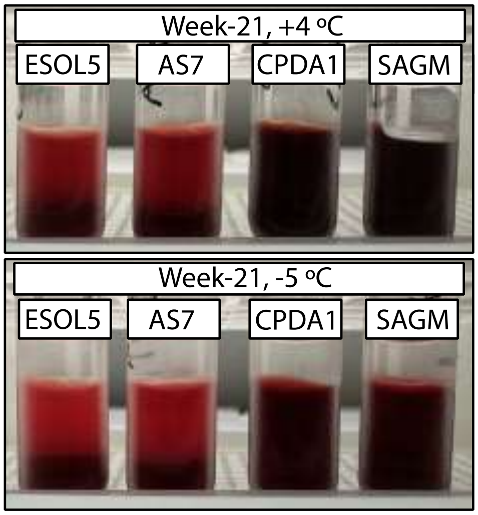

Supplementary Fig. 4 Visual comparison of E-Sol 5, AS-7, CPDA-1, and SAGM for hemolysis at +4 °C and -5 °C. **a.** The samples were stored for 21 weeks. The samples stored in CPDA-1 and SAGM experienced complete hemolysis at both temperatures. Observation was made from 2 biological replicates (N=2) and three technical replicates (n=3) for each biological replicate. Each biological replicate was from a pool of 3 donor samples.

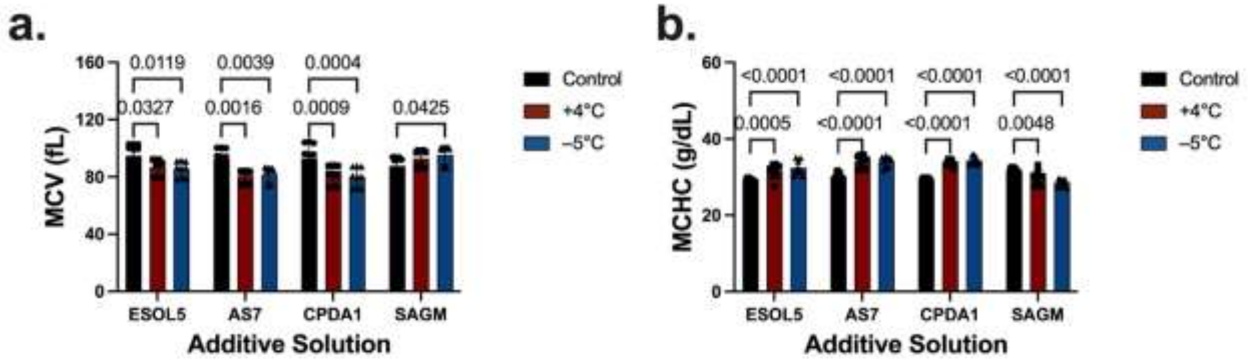

Supplementary Fig. 5 Comparison of E-Sol 5, AS-7, CPDA-1, and SAGM for mean cell volume (MCV) and mean corpuscular hemoglobin concentration (MCHC) at hypothermic (+4 °C) and supercooled (-5 °C) conditions at week-6 relative to the initial levels. The samples were stored for 6 weeks. Control represents the day-1 levels for each group. Data represent mean ± standard deviation from three biological replicates (N=3) and three technical replicates (n=3) for each biological replicate. Each biological replicate was from a pool of 3 donor samples. A two-way analysis of variance (ANOVA) followed by Tukey's *post hoc* test was performed to evaluate significant differences between conditions: *p < 0.05; **p < 0.01; ***p < 0.001; ****p < 0.0001.

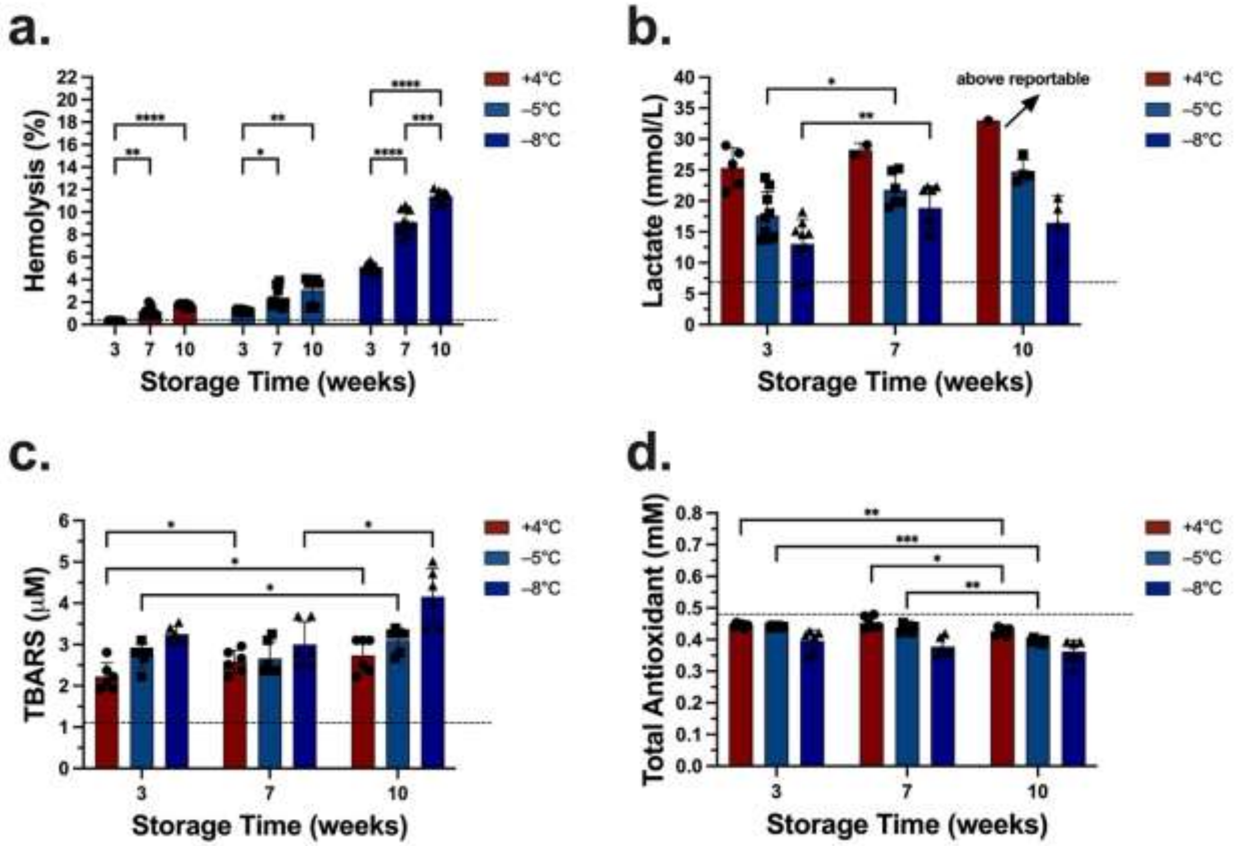

Supplementary Fig. 6 Comparison of storage performance in E-Sol 5 at 3 different temperatures. **a.** Hemolysis increased with increasing time of storage and decreasing temperature. Levels were higher at -8 °C. **b.** Lactate concentrations increased during storage and were inversely proportional to temperature. **C.** TBARS, *i.e.*, lipid peroxidation, levels were higher at -8 °C. **d.** TAC levels decreased during storage at each temperature. Dashed lines represent the mean day-1 levels following 3x washing, overnight storage at +4 °C, and final 1x washing in respective solutions. Data represent mean ± standard deviation from 3 biological replicates (N=3) and 1-3 technical replicates for each biological replicate (n=3 for hemolysis, n=2 for TBARS and TAS, n=1-3 for lactate). Each biological replicate was from a pool of 3 donor samples. A two-way analysis of variance (ANOVA) followed by Tukey's *post hoc* test was performed to evaluate significant differences between conditions: *$p < 0.05$; **$p < 0.01$; ***$p < 0.001$; ****$p < 0.0001$. Comparisons were shown across the time points at each temperature. TBARS: Thiobarbituric Acid Reactive Substances. TAC: Total Antioxidant Capacity.

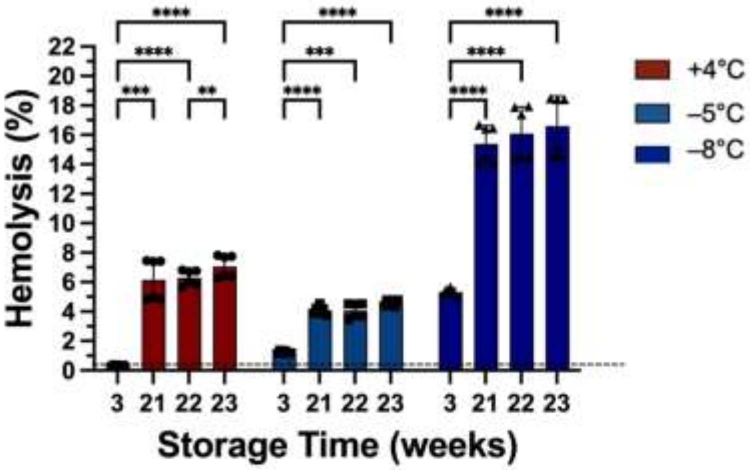

Supplementary Fig. 7 Long-term comparison of storage performance in E-Sol 5 at +4 °C and -5 °C.. At week-21 and later time points, the hemolysis for the samples stored at +4 °C was significantly higher than the hemolysis for the samples stored at -5 °C. Dashed line represents the mean day-1 levels following 3x washing, overnight storage at +4 °C, and final 1x washing in E-Sol 5. Data represent mean ± standard deviation from 2 biological replicates (N=2) and 3 technical replicates (n=3) for each biological replicate. Each biological replicate was from a pool of 3 donor samples. A two-way analysis of variance (ANOVA) followed by Tukey's *post hoc* test was performed to evaluate significant differences between conditions: *p < 0.05; **p < 0.01; ***p < 0.001; ****p < 0.0001. Comparisons were shown across the time points at each temperature.

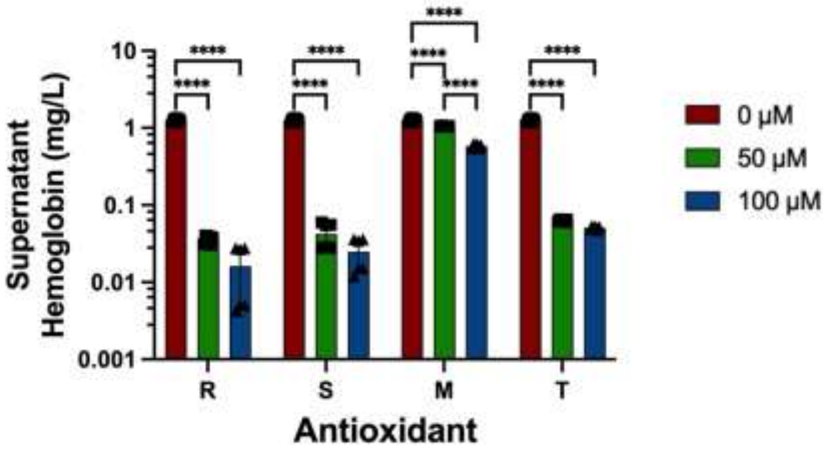

Supplementary Fig. 8 Antihemolytic effects of 4 antioxidants in response of 500 µM cumene hydroperoxide (CumOOH) treatment. RBC samples with 1% hematocrit were treated with 500 µM CumOOH in the absence (positive control, red bars) or presence of antioxidants for 3 h at +37 °C. The antioxidants were resveratrol (R), serotonin (S), melatonin (M), and Trolox (T). The concentrations supplemented were either 50 or 100 µM. Data

represent mean ± standard deviation from 2 biological replicates (N=2) and three technical replicates (n=3) for each biological replicate. Each biological replicate was from a pool of 3 donor samples. A two-way analysis of variance (ANOVA) followed by Tukey's *post hoc* test was performed to evaluate significant differences between conditions: $*p < 0.05$; $**p < 0.01$; $***p < 0.001$; $****p < 0.0001$.
